# Examining the Association Between Adverse Parenting Behaviour and Anterior Pituitary Gland Volume Development

**DOI:** 10.1192/bjo.2025.10164

**Published:** 2025-06-20

**Authors:** Julia Kim, Elena Pozzi, Sarah Whittle

**Affiliations:** 1 College of Medicine and Public Health, Flinders University, Bedford Park, Australia; 2 Department of Psychiatry, The University of Melbourne, Parkville, Australia; 3 Orygen, Melbourne, Australia.; 4 Centre for Youth Mental Health, The University of Melbourne, Melbourne, Australia

## Abstract

**Aims:** Adverse parenting behaviours (APB) are considered to contribute to the risk of depression and other psychopathologies in young people via changes in the development of the neuroendocrine stress response, particularly of the hypothalamus-pituitary-adrenal (HPA) axis. Anterior pituitary gland volume (aPGV) is emerging as a more stable biomarker of HPA axis dysregulation in comparison to cortisol measures. Although enlarged aPGV is generally understood as being reflective of chronic HPA axis hyperactivation in response to prolonged stress, there is little research that has explored the APB-aPGV relationship. Notably, there are inconsistent findings regarding longitudinal associations between APB and measures of HPA axis function (including aPGV), and it remains unclear whether exposure to APB may result in: 1) accelerated or 2) attenuated HPA axis function during childhood and adolescence. This study aims to investigate the cross-sectional and longitudinal associations between APB and aPGV in late childhood to early adolescence, in the largest sample that has been used to date.

**Methods:** Participants comprised 268 children and early adolescents from the community, who participated in longitudinal brain imaging and parenting assessments over two waves (in 8–13-year-old children). aPGV was calculated from T1-weighted Magnetic Resonance Imaging (MRI) scans of the children. APB was measured through two parent-report questionnaires. Exploratory factor analysis was used to reduce the subscales of the questionnaire to a three-factor structure; the factors were named neglect, low levels of positive parenting, and maladaptive discipline. Multiple linear regression was used to investigate cross-sectional associations between APB and aPGV, and linear mixed modelling was used to examine longitudinal associations between APB and aPGV.

**Results:** Neglect was positively associated with greater aPGV both cross-sectionally at baseline and across ages 8–13. Age did not moderate the association between neglect and aPGV longitudinally, which was stable over time. Other parenting variables were not significantly associated with aPGV changes.

**Conclusion:** Our findings suggest a crucial role for the experience of neglect in the development of the HPA axis during late childhood and early adolescence, supporting theories of HPA axis hyperactivation. The effect of neglect on aPGV was stable across age, suggesting that neglect may lead to advanced aPGV development, with accelerated development potentially occurring earlier in childhood. Further research that investigates the APB-aPGV relationship in a broader age range (i.e., covering the period between early childhood and late adolescence) is needed to understand developmental trajectories of aPGV in the context of APB.

